# Anti-Inflammatory and Free Radial Scavenging Activities of the Constituents Isolated from *Machilus zuihoensis*

**DOI:** 10.3390/molecules16119451

**Published:** 2011-11-10

**Authors:** Yi-Wen Mao, Hsiang-Wen Tseng, Wen-Li Liang, Ih-Sheng Chen, Shui-Tein Chen, Mei-Hsien Lee

**Affiliations:** 1 School of Pharmacy, College of Pharmacy, Taipei Medical University, Taipei 110, Taiwan; Email: cutemaomao2007@gmail.com (Y.-W.M.); wenlee@tmu.edu.tw (W.-L.L.); 2 Graduate Institute of Pharmacognosy, College of Pharmacy, Taipei Medical University, Taipei 110, Taiwan; 3 Biomedical Technology and Device Research Laboratories, Industrial Technology Research Institute, Hsinchu 300, Taiwan; Email: tsenghw@gmail.com (H.-W.T.); 4 Graduate Institute of Pharmaceutical Sciences, College of Pharmacy, Kaohsiung Medical University, Kaohsiung 807, Taiwan; Email: m635013@kmu.edu.tw (I.-S.C.); 5 Institute of Biological Chemistry, Academia Sinica, Taipei 115, Taiwan; 6 Institute of Biochemical Sciences, College of Life Science, National Taiwan University, Taipei 106, Taiwan; 7 Center for Reproductive Medicine & Sciences, Taipei Medical University Hospital, Taipei 110, Taiwan

**Keywords:** *Machilus zuihoensis*, anti-inflammatory, quercetin-3-*O*-β-D-glucopyranoside-(3′→*O*-3‴)-quercetin-3-*O*-β-D-galactopyranoside, ethyl caffeate, quercetin

## Abstract

A new biflavonol glycoside, quercetin-3-*O*-β-D-glucopyranoside-(3′→*O*-3‴)- quercetin-3-*O*-β-D-galactopyranoside (**9**), together with eight known compounds was isolated for the first time from the leaves of *Machilus zuihoensis* Hayata (Lauraceae). The structure of compound **9** was elucidated by various types of spectroscopic data analysis. Analysis of the biological activity assay found that compound **9** showed significant superoxide anion scavenging activity (IC_50_ is 30.4 μM) and markedly suppressed LPS-induced high mobility group box 1 (HMGB-1) protein secretion in RAW264.7 cells. In addition, the HMGB-1 protein secretion was also inhibited by quercitrin (**3**), ethyl caffeate (**6**), and ethyl 3-*O*-caffeoylquinate (**7**) treatment. In the LPS-stimulated inducible nitric oxide synthase (*i*NOS) activation analysis, two known compounds, quercetin (**1**) and ethyl caffeate (**6**), were found to markedly suppress nitric oxide (NO) production (IC_50_ value, 27.6 and 42.9 μM, respectively) in RAW264.7 cells. Additionally, it was determined that ethyl caffeate (**6**) down-regulated mRNA expressions of *i*NOS, IL-1β, and IL-10 in the LPS-treatment of RAW264.7 cells via a suppressed NF-κB pathway. These results suggested for the first time that the new compound **9** and other constituents isolated from *M. zuihoensis* have potential anti-inflammatory and superoxide anion scavenging effects. These constituents may be useful for treating various inflammatory diseases.

## 1. Introduction

Reactive oxygen free radical species (ROS) induce oxidative stress, which causes a wide variety of pathological effects, such as DNA damage and cellular degeneration related to aging [1] and diseases [2], including inflammatory diseases, cancer, and neurodegenerative diseases. Some significant inflammatory roles of O_2_^•−^ include: increasing vascular permeability, enhancement of pro-inflammatory cytokines (e.g., TNF-α, IL-8, and IL-1β) and chemotactic factor (e.g., leukotriene B4) formation, as well as recruitment of neutrophils at sites of inflammation [3,4]. Chronic and acute inflammation is a complex process mediated by activating inflammatory or immune cells [5,6]. During inflammation, macrophages play a central role in mediating many different immunopathological phenomena, such as the overproduction of pro-inflammatory cytokines and inflammatory mediators, such as IL-1β, IL-6, nitric oxide (NO), *i*NOS, tumor necrosis factor-α (TNF-α), and high mobility group box 1 (HMGB-1) [6,7]. The HMGB-1 protein is an abundant protein in nuclei and cytoplasm that is involved in maintaining nucleosome structure and facilitates gene transcription [8,9]. Recent studies suggest that HMGB-1 proteins released from the nucleus of activated macrophages or necrotic cells act as endogenous immune-adjuvants through activation of immune cells, including dendritic cells, macrophages, and T cells [8,9,10]. Therefore, HMGB-1 is a necessary and sufficient mediator in severe sepsis because independent strategies that inhibit its inflammatory potential prevent multiple organ failure and rescue mice from established sepsis [11,12].

In the study of plants as sources of natural physiologically active substances, medicinal plants, vegetables, and fruits have been extensively studied for their anti-oxidant, anti-inflammatory and anti-cancer activities in the last few years [13,14,15,16]. *Machilus zuihoensis* Hayata (Lauraceae) is an evergreen species endemic to Taiwan. Its bark is one of the materials used to make incense sticks. In addition, its wood is used for making bridges, ships and furniture [17]. In a previous paper, some compounds (steryl epoxide, secobutanolide, butanolides, benzenoids and steroids) were isolated from the stem wood of *M. zuihoensis*. The stem wood of *M. zuihoensis* showed cytotoxic activity against NUGC-3 and HONE-1 cancer cell lines *in vitro* [18]. We have previously reported that a 95% EtOH extract of the leaves of *M. zuihoensis* showed significant free-radical scavenging activity [19]. To identify the active constituents from the leaves of *M. zuihoensis*, several isolation methods were used to purify the constituents and to evaluate their anti-inflammatory and free radical scavenging activities. 

## 2. Results and Discussion

**Figure 1:**
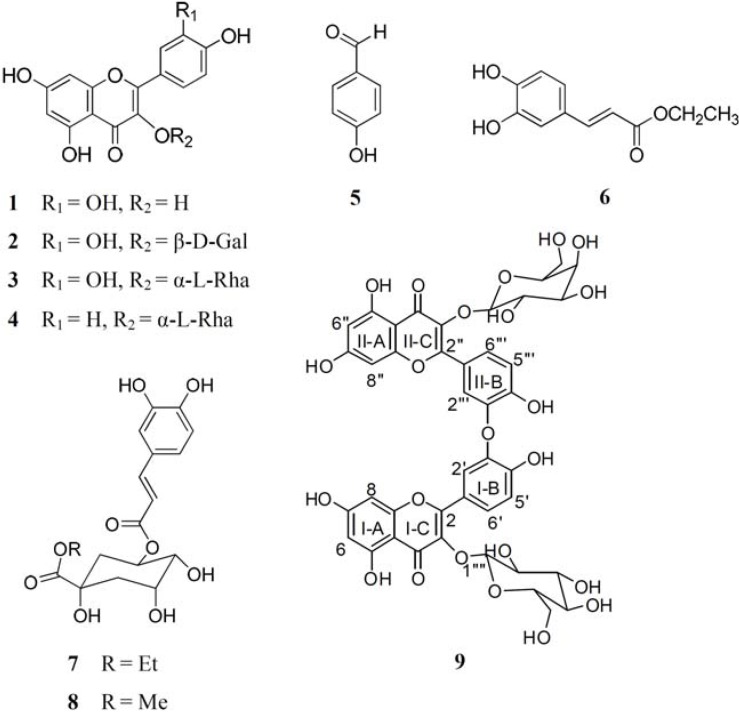
Chemical structures of compounds **1–9**.

An ethanol extract of the leaves of *M. zuihoensis* yielded one new compound (compound **9**) along with eight known compounds, including four flavonols: quercetin (**1**) [20], hyperoside (**2**) [21], quercitrin (**3**) [22], and afzelin (**4**) [20]; one phenyl derivative, 4-hydroxybenzaldehyde (**5**) [23], as well as three caffeoyl derivatives, ethyl caffeate (**6**) [24], ethyl 3-*O*-caffeoylquinate (**7**) [25], and clorogenic acid methyl ester (**8**) [26]. Structural determination of new compound **9** is described in the Experimental Section. Other known compounds were identified by comparison of their spectral data (UV, ^1^H-NMR, ^13^C-NMR, ESI-MS) with the corresponding data in the literature or with authentic samples. The chemical structures of these compounds are shown in Figure 1.

### 2.1. Effects of the Constituents of Leaves of *M. zuihoensis* on Superoxide Anion Radical (O_2_^•−^) Scavenging Activities

Antioxidant activity is important in view of the free radical theory of aging and associated diseases [2]. In the present study, superoxide anion radical scavenging activities of isolated constituents were evaluated by the Nitro Blue Tetrazolium (NBT) assay. The plant constituent, (+)-catechin, was used as the positive control (IC_50_ = 41.6 μM). Five of the isolated constituents, namely quercetin (**1**), quercitrin (**3**), ethyl caffeate (**6**), clorogenic acid methyl ester (**8**), and quercetin-3-*O*-β-D-glucopyranoside-(3′→*O*-3‴)-quercetin-3-*O*-β-D-galactopyranoside (**9**), showed dose-dependent activity and their IC_50_ values were 42.6, 75.1, 66.7, 99.0 and 30.4 μM, respectively (Table 1).

**Table 1:** Superoxide anion radical (O_2_^•−^) scavenging activity of constituents isolated from *M. zuihoensis.*

Sample	IC_50_ (μM)
(+) Catechin (positive control)	41.6
Quercetin (**1**)	42.6
Hyperoside (**2**)	>100
Quercitrin (**3**)	75.1
Afzelin (**4**)	>100
4-Hydroxybenzaldehyde (**5**)	>100
Ethyl caffeate (**6**)	66.7
Chlorogenic acid ethyl ester (**7**)	>100
Clorogenic acid methyl ester (**8**)	99.0
Quercetin-3-*O*-β-D-glucopyranoside-(3′→*O*-3‴)-quercetin-3-*O*-β-D-galactopyranoside (**9**)	30.4

Structural and functional correlation of some flavonoids for superoxide anion scavenging activity has been demonstrated in previous studies. The existence of the double bond between C-2 and C-3, OH group at C-3 and C-5, and the keto group at C-4 are important features for superoxide scavenging activity [27,28]. The new compound **9** showed potent scavenging activity which may be due to the fact it possesses these characteristics.

### 2.2. Effects of Constituents of Leaves of *M. zuihoensis* on Anti-Inflammatory Activities

**Figure 2:**
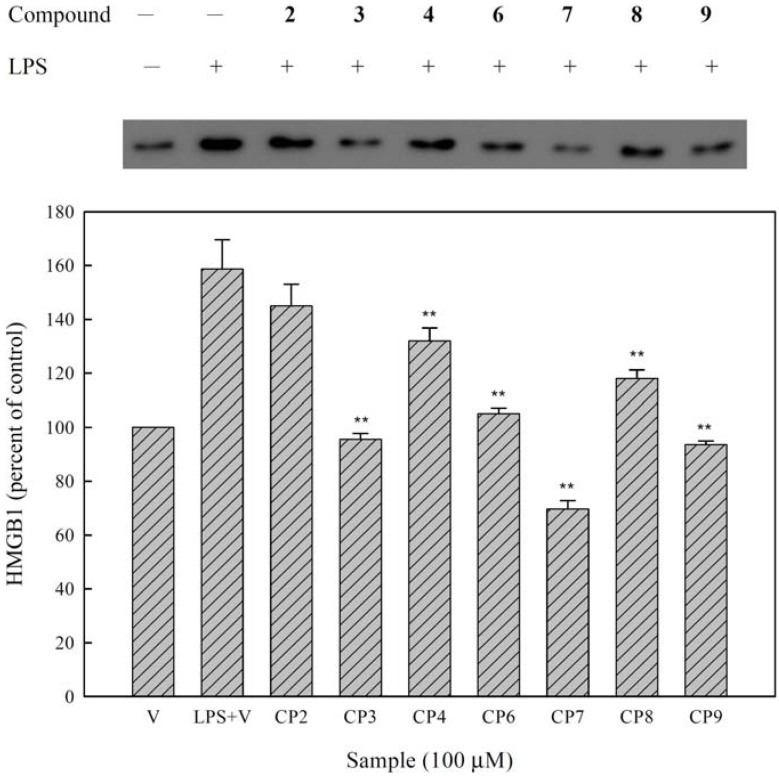
Effects of the constituents of leaves from *M. zuihoensis* on LPS-induced HMGB-1 protein secretion in RAW264.7 cells. Cells were treated with compounds **2**–**4**, **6**–**9** (100 μM) for 2 hours followed by the addition of LPS (20 ng/mL^−1^). Levels of HMGB-1 in the culture medium were determined by Western blot analysis at 30 hours after LPS stimulation. The statistical analyses for LPS + V and compounds treatment were performed using student’s t test. Significant inhibition is indicated by **, with a *P*-value < 0.01.

A number of inflammatory stimuli, such as LPS and proinflammatory cytokines (e.g., TNF-α), activate immune cells to up-regulate inflammatory states [29]; therefore, they represent useful targets for developing new anti-inflammatory constituents and exploring their molecular mechanisms [30]. HMGB-1 is secreted by macrophages activated with LPS or proinflammatory cytokines and induced with LPS or proinflammatory mediators from these cells [9,31]. Unlike other proinflammatory cytokines (e.g., TNF-α), HMGB-1 is a late-appearing inflammatory mediator; consequently, it provides a wider time frame for clinical intervention against progressive inflammatory disorders [11]. In contrast to other proinflammatory cytokines, HMGB-1 is secreted from macrophages approximately 20 hours post-stimulation [32,33]. Therefore, we further assessed the effects of constituents isolated from the leaves of *M. zuihoensis* on HMGB-1 protein secretion in LPS-stimulated RAW264.7 cells (Figure 2). Western blot analysis of the cell culture supernatant revealed that LPS caused an increase in HMGB-1 protein secretion compared with the control group. The results showed that new compound quercetin-3-*O*-β-D-glucopyranoside-(3′→*O*-3‴)-quercetin-3-*O*-β-D-galactopyranoside (**9**) and three other compounds containing quercitrin (**3**), ethyl caffeate (**6**), and ethyl 3-*O*-caffeoylquinate (**7**), significantly inhibited the LPS-induced HMGB-1 protein secretion.

**Figure 3:**
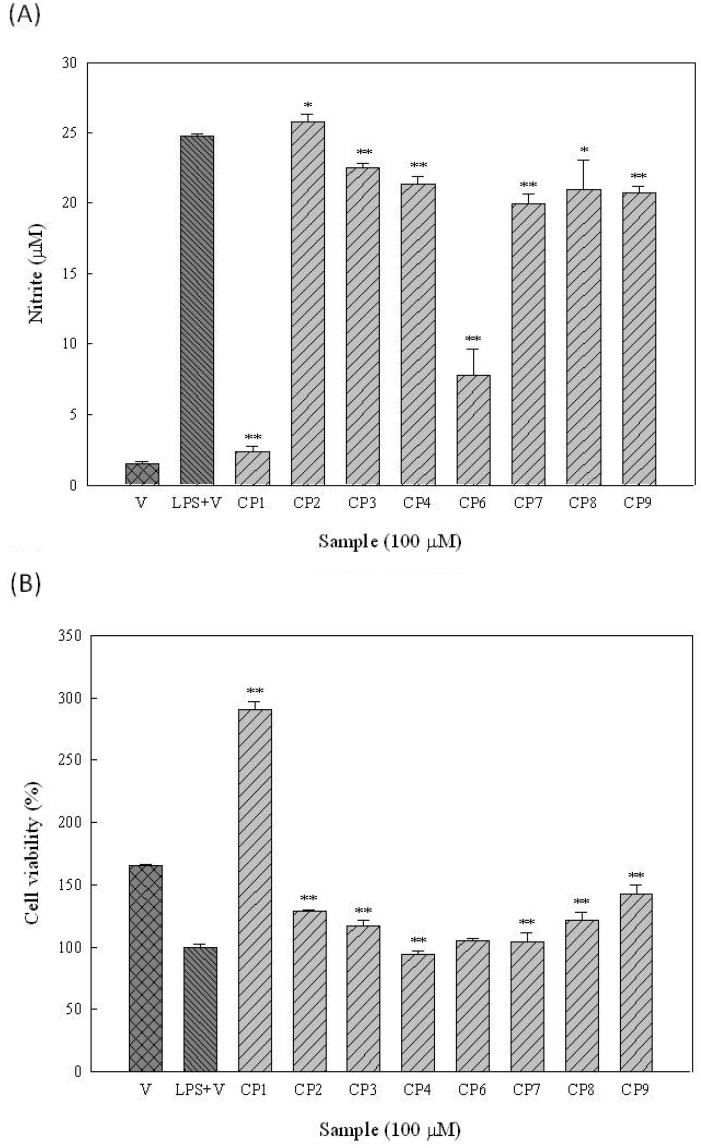
Effects of constituents of leaves of *M. zuihoensis* on nitrite formation (**A**) and cell viability (**B**) in RAW264.7 macrophages. RAW264.7 macrophages were cultured at 37 °C for 24 hours in a 24-well plate in the presence of vehicle (V, DMSO), LPS (20 ng/mL) in combination with indicated concentrations of compounds (CP). The culture supernatant was mixed with Griess reagent for nitrite analysis. Otherwise, cell viability was determined using the AlamarBlue assay. Data present the mean ± SD. The statistical analyses for LPS (**A**) or vehicle control (**B**) and compounds treatment were performed using student’s t test. Significant inhibition is indicated by * and **, with a *P*-value < 0.05 and <0.01, respectively.

Moreover, in the screening study we found that extract (20 μg/mL) of the leaf of *M. zuihoensis* significantly suppressed LPS-induced nitric oxide (NO) production with an inhibition rate of 36.2% (data not shown). The effects of the constituents isolated from the leaves of *M. zuihoensis* on nitric oxide (NO) production in LPS-stimulated RAW264.7 cells are shown in Figure 3A. Two of the compounds, namely quercetin (**1**) and ethyl caffeate (**6**), were found to suppress the LPS-induced nitric oxide (NO) production in a dose-dependent manner, with IC_50_ values of 27.6 and 42.9 μM, respectively. To further evaluate whether the observed inhibition of NO production in RAW264.7 cells was accompanied by cytotoxic effects, cell viability studies were performed using an Alamar Blue assay (Figure 3B). None of the compounds were found to cause significant cytotoxicity to the RAW264.7 cells at concentrations of 100 μM or below.

**Figure 4:**
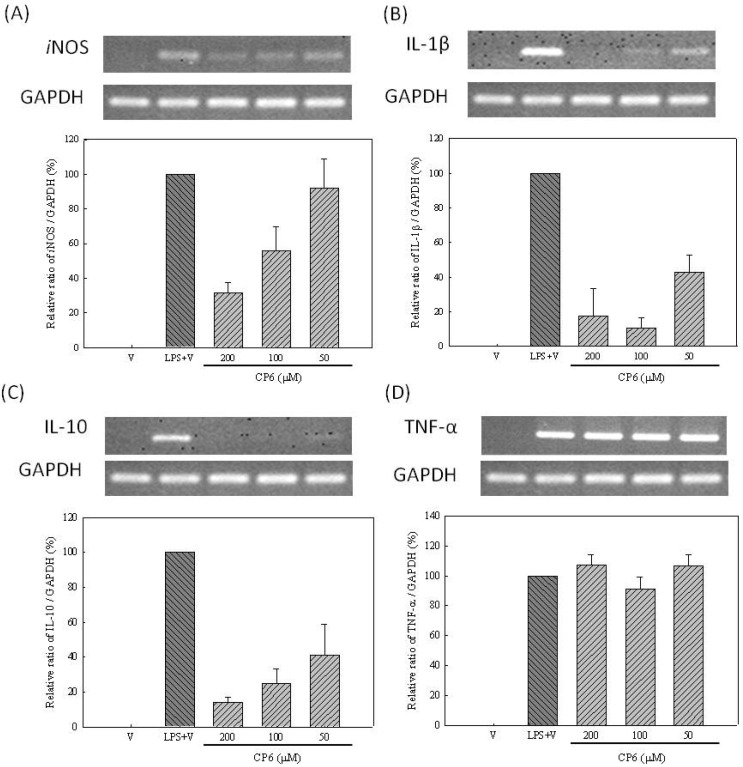
Effects of ethyl caffeate (**6**) on LPS-induced *i*NOS, IL-1β, IL-10 and TNF-α mRNA expression in RAW264.7 cells. RAW264.7 cells were pre-treated with the indicated concentrations of ethyl caffeate (**6**) for 2 hours followed by the addition of LPS (20 ng·mL^−1^) for 6 hours. Total cellular RNA (5 μg) was subjected to RT-PCR and the final PCR product was resolved using 1.5% agarose gel electrophoresis. (**A**) *i*NOS (**B**) IL-1β (**C**) IL-10 (**D**) TNF-α. Data was presented as the mean ± SD. Quantification of *i*NOS, IL-1β, IL-10 and TNF-α mRNA expression was normalized to GAPDH using a densitometer.

In previous studies, quercetin (**1**) has been reported to exhibit anti-inflammatory effects through regulation of nitric oxide and TNF-α production by NF-κB pathway in LPS-stimulated macrophages [34,35], inhibit the IL-6 release in LPS-stimulated neutrophils [36], reduce the circulating levels of HMGB1 in animals with established endotoxemia, and inhibit the LPS-induced HMGB-1 release in macrophage cultures [37]. Nevertheless, ethyl caffeate (**6**) was only found to suppress NF-κB activation and its downstream inflammatory mediators, *i*NOS, COX-2, and PGE_2_ *in vitro* [38]. Therefore, the effect of ethyl caffeate (**6**) on IL-1β, IL-10, and TNF-α mRNA expression levels that mediate the synthesis of NO and cytokine in LPS-stimulated RAW264.7 cells was further assessed. RT-PCR analysis of the extracted RNA revealed that LPS caused an increase in *i*NOS, IL-1β, IL-10, and TNF-α mRNA expression compared with the control group, indicating that ethyl caffeate (**6**) significantly inhibited LPS-induced *i*NOS, IL-1β, and IL-10 mRNA expression in a dose-dependent manner, but that it did not inhibit TNF-α mRNA expression (Figure 4).

The activation of the SAPK/JNK, p38, and p42/44 (ERK 1/2) MAPK proteins, known to be involved in the regulation of *i*NOS or expression of other pro-inflammatory cytokines, was further investigated to determine if there was any link between these signaling molecules and the anti-inflammatory mechanism of ethyl caffeate (**6**). Pathway inhibitors, such as SB203580 (p38 inhibitor, 10 μM), Wortmannin (PI_3_K/Akt inhibitor, 1 μM), SP600125 (JNK inhibitor, 10 μM), U0126 (ERK inhibitor, 10 μM), and PDTC (NF-κB inhibitor, 100 μM) pre-treated with RAW264.7 cells were utilized. The effects of the PDTC inhibitor on LPS-induced *i*NOS and the expression of other pro-inflammatory cytokine mRNA were similar to those of ethyl caffeate (**6**) (Table 2). Therefore, it was demonstrated that ethyl caffeate (**6**), *via* the NF-κB pathway, down regulated mRNA expression of *i*NOS, IL-1β, and IL-10 production in RAW264.7 cells.

**Table 2:** Effects of compound **6** and five different blockers on LPS-induced *i*NOS, IL-1β, TNF-α, IL-10 mRNA expression in RAW264.7 cells.

Samples	*i*NOS	IL-1β	TNF-α	IL-10
Ethyl caffeate (**6**)	↓	↓	─^ a^	↓
SB203580 (p38 inhibitor, 10 μM)	─ ^a^	↓	─^ a^	↓
Wortmannin (PI_3_K/Akt inhibitor, 1 μM)	─ ^a^	─^ a^	─^ a^	─^ a^
SP600125 (JNK inhibitor, 10 μM)	↓	↓	↓	↓
U0126 (ERK inhibitor, 1 μM)	─^ a^	↓	↓	↓
PDTC (NF-κB inhibitor, 100 μM)	↓	↓	─^ a^	↓

^a^ Means no significant effect.

During the progression of the inflammatory diseases, pro-inflammatory early cytokines (such as TNF-α and IL-1β) are typically produced within minutes of stimulation and revert to near-baseline levels within the first few hours [12,39]. HMGB1 is a late mediator of inflammatory diseases. Macrophages secrete HMGB1 after 20 hours activation and serum HMGB1 is detected in a prolonged plateau beginning 20–72 hours after the onset of the diseases [8,12]. In the previous studies, quercetin (**1**) showed inhibitory effects on both early (e.g., *i*NOS, IL-1β, TNF-α and IL-10) [34,35,36] and late (e.g., HMGB-1) [37] inflammatory responses after LPS induction. Ethyl caffeate (**6**) was found to suppress NF-κB activation and its downstream early inflammatory mediators, *i*NOS, COX-2, and PGE_2_ *in vitro* or in mouse skin [38]. In the present study, we demonstrated that ethyl caffeate (**6**) exhibits the capacity to regulate early inflammatory mediator mRNA expression of *i*NOS, IL-1β, and IL-10 (Figure 4) and late (HMGB-1) inflammatory mediator protein production (Figure 2) in LPS-induced RAW264.7 cells. Quercitrin (**3**), ethyl 3-*O*-caffeoylquinate (**7**) and the new compound quercetin-3-*O*-β-D-glucopyranoside-(3′→*O*-3‴)-quercetin-3-*O*-β-D-galactopyranoside (**9**) exhibit inhibitory effects of the late inflammatory mediator HMGB-1 protein secretion. Some previous reports demonstrated that LPS induced the release of HMGB-1 and was associated with the TLR4, TRIF, IFN-β, and JAK-STAT pathways [40]. Therefore, compounds **3**, **7**, and **9** may have suppressed the late inflammatory response *via* these inflammatory pathways in different ways compared to compounds **1** and **6**. Consequently, these constituents isolated from *M. zuihoensis* could have suppressed both acute and chronic LPS-induced inflammatory responses and may lead to the prevention of lethal systemic inflammation.

## 3. Experimental

### 3.1. General

Optical rotations were measured on a JASCO P-1020 digital polarimeter. UV spectra were recorded on a Hitachi U-2800 UV/vis spectrometer. 1D-and 2D-nuclear magnetic resonance (NMR) spectra were measured with a Bruker AM-500 spectrometer using acetone-*d*_6_, MeOH-*d*_4_, and D_2_O solutions. HRESI-MS and ESI-MS analyses were performed with the VG Platform Electrospray ESI/MS.

### 3.2. Plant Materials

The leaves of *Machilus zuihoensis* were collected from the Highlands Experiment Farm, National Taiwan University, Nantou, Taiwan, and were identified by Mr. Chi-Luan Wen, Seed Improvement and Propagation Station, Council of Agriculture, Taiwan. A voucher specimen (M-343) was deposited in the Graduate Institute of Pharmacognosy, Taipei Medical University, Taiwan.

### 3.3. Extraction and Isolation

Fresh leaves (21.5 kg) of *M. zuihoensis* were extracted four times with aqueous EtOH (95% v/v, total 240 L). After filtration, the filtrate was evaporated and dried under vacuum. The dried extract (785.0 g) was suspended in H_2_O and successively partitioned with *n*-hexane, EtOAc, and *n*-BuOH to yield 323.0 g, 312.8 g, and 81.8 g of extract, respectively. The EtOAc extract with the greatest protective activity was subjected to an activity-guided fractionation using Diaion HP-20 column chromatography by elution with H_2_O-MeOH (1:0 to 0:1). The active fraction, MZ-1-4 (26.3 g, eluted with H_2_O-MeOH 4:6), was further separated by Sephadex LH-20 column chromatography and eluted with 95% EtOH to yield eight fractions (MZ-2-1~MZ-2-8). Fraction MZ-2-3 was fractionated by ODS-C_18_ column chromatography with H_2_O-MeOH gradient elution to yield four subfractions (MZ-3-1~MZ-3-4). Compounds **5** (2.6 mg) and **7** (26.5 mg) were obtained from the MZ-3-2 fraction after purification by preparative reversed-phase high-performance liquid chromatography (HPLC) with MeOH-H_2_O (48%) as the eluting solvent system. Compounds **2** (667.4 mg) and **3** (41.7 mg) were obtained from the MZ-2-5 fraction after recrystallization and purification by preparative reversed-phase HPLC with MeOH-H_2_O (48%) as the eluting solvent system. Another active fraction, MZ-1-5 (48.0 g, eluted with H_2_O-MeOH 2:8), was further separated by Sephadex LH-20 column chromatography and eluted with 95% EtOH to yield eleven fractions (MZ-5-1~MZ-5-11). Fraction MZ-5-3 was fractionated by ODS-C_18_ column chromatography with an H_2_O-MeOH gradient elution to yield four subfractions (MZ-6-1~MZ-6-11). Compound **6** (16.5 mg) was obtained from the MZ-6-5 fraction. Fraction MZ-5-4 was fractionated by ODS-C_18_ column chromatography with an H_2_O-MeOH gradient elution to yield fifteen subfractions (MZ-7-1~MZ-7-15). Compounds **8** (14.1 mg) and**4** (8.9 mg) were obtained from the MZ-7-2 and MZ-7-9 fractions after purification by preparative reversed-phase HPLC with MeOH-H_2_O (40%) as the eluting solvent system. Another active fraction, MZ-5-5 was fractionated by ODS-C_18_ column chromatography with an H_2_O-MeOH gradient elution to yield twelve subfractions (MZ-8-1~MZ-8-12). Compound **9** (265.3 mg) was obtained from the MZ-8-2 fraction. Another active fraction, MZ-5-8 was fractionated by ODS-C_18_ column chromatography with an H_2_O-MeOH gradient elution to yield eight subfractions (MZ-9-1~MZ-9-8). Compound **1** (11.4 mg) was obtained from the MZ-9-2 fraction after purification by preparative reversed-phase HPLC with MeOH-H_2_O (65%) as the eluting solvent system.

### 3.4. Spectral Data of the New Compound Quercetin-3-O-β-D-glucopyranoside-(3′→O-3‴)-quercetin-3-O-β-D-galactopyranoside (**9**)

Yellow brown powder, [α]^22^_D_ = −16.5 (*c* = 1.0, MeOH). UV (MeOH): 359 (4.37), 259 (4.18), 214 (4.82). ^1^H-NMR (500 MHz, acetone-*d*_6_): δ_H_ 8.00 (1H, brs, H-2‴), 7.86 (1H, brs, H-2′), 7.54 (2H, d, *J* = 8.4 Hz, H-6′ and H-6‴), 6.92 (1H, d, *J* = 8.4 Hz, H-5‴), 6.90 (1H, d, *J* = 8.4 Hz, H-5′), 6.43 (2H, brs, H-8 and H-8″), 6.20 (2H, brs, H-6 and H-6″), 5.25 (1H, d, *J* = 7.3 Hz, H-1′‴), 5.16 (1H, d, *J* = 7.3 Hz, H-1″‴), 3.96 (1H, brs, H-4″‴), 3.89 (1H, m, H-2″‴), 3.68 (2H, m, H-3‴″ and H-6″‴), 3.67 (1H, m, H-6″‴), 3.63 (1H, d, *J* = 4.8 Hz, H-6′‴), 3.57 (1H, m, H-5″‴), 3.56 (1H, m, H-5‴′), 3.55 (1H, m, H-2‴′), 3.54 (1H, m, H-6‴″), 3.47 (1H, m, H-4‴′), 3.33 (1H, m, H-3‴′). ^13^C-NMR (125 MHz, acetone-*d*_6_): δ_C_ 178.7 (C-4″), 178.6 (C-4), 165.2 (C-7 and C-7″), 162.0 (C-5″), 161.9 (C-5), 158.3 (C-2), 157.9 (C-2″), 157.6 (C-9″), 157.5 (C-9), 149.2 (C-4‴), 149.1 (C-4′), 145.0 (C-3′ and C-3‴), 135.1 (C-3″), 135.0 (C-3), 122.5 (C-6′ and C-6‴), 122.2 (C-1′), 122.1 (C-1‴), 117.6 (C-2‴), 117.4 (C-2′), 115.7 (C-5′ and C-5‴), 104.9 (C-10 and C-10″), 104.8 (C-1‴″), 104.0 (C-1‴′), 99.5 (C-6 and C-6″), 94.5 (C-8 and C-8″), 77.3 (C-5‴′), 77.2 (C-3‴′), 76.2 (C-5‴″), 74.9 (C-2‴′), 74.3 (C-3‴″), 72.5 (C-2‴″), 70.2 (C-4‴′), 68.9 (C-4‴″), 61.8 (C-6‴′), 61.0 (C-6‴″). HR-ESI-MS: 951.1792 ([*M*+K+2H]^+^, C_42_H_40_KO_23_^+^; calc. 951.1597).

#### 3.4.1. Acid Hydrolysis of Compound 9

One milligram of compound **9** was hydrolyzed with 6 N HCl at 80 °C in a heating block for 6–8 hours. The mixture was cooled and evaporated to remove the acid, then resuspended in milli-Q water and passed through a Millipore-GX nylon membrane before analysis [41]. Monosaccharides of compound **9** and polysaccharide hydrolysates were separated on a high-performance anion-exchange chromatographic (HPAEC) system (Dionex, Sunnyvale, CA, USA) and an anion-exchange column (Carbopac PA-10, 4.6 × 250 mm). The analysis of the monosaccharides was carried out using an isocratic 18 mM NaOH solution at ambient temperature.

#### 3.4.2. Structural Determination of Compound 9

The ^1^H-NMR and ^13^C-NMR spectra, together with UV absorption at λ_max_ 359, 259 and 214 nm revealed that compound **9** possessed two flavone moieties. The presence of signals at δ_H_ = 7.86 (1H, br s), 6.90 (1H, d, *J* = 8.4 Hz), 7.54 (2H, d, *J* = 8.4 Hz), 8.00 (1H, br s), and 6.92 (1H, d, *J* = 8.4 Hz) in an ABX coupling system, together with the signals of δ_c_ = 117.4, 117.6, 115.7, and 122.5 observed in the NMR spectra suggested two B-ring (Ι and ΙΙ) aromatic moieties in compound **9**. In the Heteronuclear Multiple Bond Correlation (HMBC) spectrum, two aromatic signals, δ_H_ = 6.20 (2H, br s) and 6.43 (2H, br s), were assigned at H-6, H-6″ and H-8, H-8″, respectively, due to the long-range coupling with C-5, 5″, 7, 7″, 8, 8″ and C-6, 6″, 7, 7″, 9, 9″, respectively. The NMR spectrum also showed two sugar signals δ_H_ = 3.33 to 5.25 and δ_C_ = 61.0 to 104.8. After the acid hydrolysis of compound **9**, the aqueous layer was separated by HPLC to yield two glycosides: glucose and galactose. The position of the sugar linkage was confirmed at C-3 and C-3″ by HMBC correlations. The configurations of the anomeric protons of **9** were both deduced to be β form on the basis of the coupling constants [H-1‴′ (*J* = 7.3 Hz) and H-1‴″ (*J* = 7.8 Hz)]. These spectral studies suggested that compound **9** could be a biflavonol glycoside consisting of two flavonol groups and two sugar moieties with an ether linkage. The ether linkage of the two flavonol groups was confirmed by the positive Nuclear Overhauser Effect (NOE) between H-2′ (δ_H_ = 7.86) and H-2‴ (δ_H_ = 8.00); thus it was suggested that the C-3′ of ring B-Ι was involved in the interflavonoid ether linkage with the C-3″ of ring B-ΙΙ. Based on the aforementioned data, compound **9** was determined to be a biflavonol glycoside, quercetin-3-*O*-β-D-glucopyranoside-(3′→*O*-3‴)-quercetin-3-*O*-β-D-galactopyranoside (Figure 1).

### 3.5. Reagents

Dulbecco’s Modified Eagle’s Medium (DMEM), Hanks’ balanced salt solution (HBSS), phosphate-buffered saline (PBS), DMSO (dimethyl sulfoxide), LPS (*Escherichia coli* 0111:B4), Nitro blue tetrazolium (NBT), xanthine, xanthine oxidase, *N*-(1-naphthyl) ethylenediamine dihydrochloride (NEDD), sulfanilamide, and agarose were purchased from Sigma-Aldrich (Sigma, St. Louis, MO, USA). Fetal bovine serum (FBS) was purchased from Gibco (Gibco Canada Inc., Burlington, ON, Canada). All chemicals and reagents used in the study were high-grade commercial products. Other materials included a High Pure RNA Isolation Kit (Roche, Mannheim, Germany), SuperScript^TM^ II reverse transcriptase (Invitrogen Inc., Burlington, ON, Canada), and AlamarBlue reagent (Alamar Biosciences, Inc., Sacramento, CA, USA).

### 3.6. NBT (Superoxide Scavenging) Assay

Superoxide anion radicals were generated by the xanthine/xanthine oxidase system and monitored with the product NBT, using a modification of the described procedure [42]. The samples were dissolved in phosphate buffer 100 mM (pH 7.4). The reaction mixtures contained different concentrations of test samples, 100 μM of xanthine, and 500 μM of NBT. The reaction mixtures were incubated at ambient temperature for 2 min and the reaction was initiated with the addition of 15 mU xanthine oxidase. The color reaction of the superoxide radicals with NBT was detected at OD 560 nm and (+)-catechin was used as a positive control. The inhibition ratio (%) was calculated from the following equation: % inhibition = [(absorbance of control – absorbance of test sample)/absorbance of control] × 100%.

### 3.7. Cell Culture

RAW264.7 cells (murine macrophage-like cell line) were obtained from Bioresource Collection and Research Center. RAW264.7 cells were cultured at 37 °C in a 5% CO_2_ atmosphere in DMEM medium. The medium was supplemented with 10% fetal bovine serum (FBS). RAW264.7 cells were used for NO production, cell viability, cytokine mRNA expression, and HMGB-1 protein secretion assays.

### 3.8. Western Blot Assay for HMGB-1

The levels of HMGB-1 in the culture medium were determined by Western blot analysis as previously described [43,44]. Equal volumes of cell culture supernatant were mixed with 4× sample buffer (30% glycerol, 2% β-mercaptoethanol, 8% SDS, 0.25 M Tris-HCl, pH 6.8, and 0.4% bromphenol blue). After boiling for 5 min, the samples were subjected to a 12.5% SDS-polyacrylamide gel electrophoresis. The resolved proteins were transferred to a PVDF membrane (Millipore Corporation, Billerica, MA) and blocked with a blocking buffer [1 × phosphate- buffered saline (PBS), 5% nonfat milk, and 0.05% Tween 20] for 2 hours. The membrane was then incubated with rabbit polyclonal anti-HMGB-1 antibodies (Abcam, Cambridge, UK) and diluted in a blocking buffer (1:5000) for 2 hours. After washing three times in 1× PBST buffer, immunoblots were exposed to room temperature for 2 hours and diluted to a 1:2,000 dilution with horseradish peroxidase-conjugated goat anti-rabbit secondary antibody (Abcam). After washing three times with 1 × PBST buffer, the membrane was illuminated with ECL reagent (Amersham Life Sciences, Little Chalfont, UK) and the X-ray film was exposed, according to the manufacturer’s instructions.

### 3.9. AlamarBlue Assay and Measurement of NO

RAW264.7 cells were seeded in 24-well plates at a density of 3 × 10^5^ cells well^−1^. Twenty-four hours after plating, the cells were treated with test compounds for 2 hours and then incubated for 24 hours in fresh DMEM with or without 20 ngmL^−1^ of LPS. Cell viability was determined using the AlamarBlue assay as described elsewhere [45]. Nitrite (a stable oxidative end product of NO) accumulation in the medium of RAW264.7 cells was determined by the Griess reaction [46]. Briefly, 100 μL cell culture supernatants were reacted with 100 μL of Griess reagent (1:1 mixture of 0.1% *N*-(1-naphthylethylene)diamine dihydrochloride (NEDD) in H_2_O and 1% sulfanilamide in 5% phosphoric acid) in a 96-well plate, and the absorbance at 570 nm was recorded using an ELISA reader.

### 3.10. RNA Extraction and RT-PCR

The RAW264.7 cell line was treated with compound **6** (20, 50, 100 and 200 μM) for 2 hours and then incubated for 6h in fresh DMEM with or without 20 ngmL^−1^ of LPS. The total RNA was extracted with a High Pure RNA Isolation Kit (Roche, Mannheim, Germany) and was reverse transcribed with SuperScript ^TM^ II reverse transcriptase, according to the manufacturer’s instructions. The sequences of the oligonucleotides used in the PCR reactions were as follows: GAPDH, 5′-TGGGTGTGAACCACGA GAAA-3′ (forward) and 5′-GGTCATGAGCCCTTCCACAA-3′ (reverse); IL-1β, 5′-TCGCTCAGGG TCACAAGAAA-3′ (forward) and 5′-CTGCCTAATGTCCCCTTGAATC-3′ (reverse); IL-10, 5′-CTTG CAGAAAAGAGAGCTCCA-3′ (forward), and 5′-TTGATTTCTGGGCCATGCTTC-3’ (reverse); TNF-α, 5′-TCCCCAAAGGGATGAGAAGTTC-3′ (forward) and 5′-TCATACCAGGGATGAGAAGTTC-3′ (reverse); *i*NOS, 5′-GCCCTGCTTTGTGCGAAGT-3′ (forward) and 5′-GCTCATGCGGCCTCCTTT-3′ (reverse). The resulting products were analyzed by electrophoresis on 1.5% agarose gels and stained with ethidium bromide.

### 3.11. Statistical Analysis

Student’s t test was used for determining the statistically significant differences between the values of various experimental groups. Data were expressed as means ± SD and a *P*-value < 0.05 was considered statistically significant.

## 4. Conclusions

Reactive oxygen species, the consequence of an imbalance of prooxidants and antioxidants in the organism, is gaining recognition as a key phenomenon in chronic illnesses like inflammatory disease, cancer, and atherosclerosis. Moreover, several studies suggest that antioxidant and anti-inflammatory agents could be beneficial in the prevention and treatment of these pathologies [47,48]. Consequently, this is the first reported article showing the anti-inflammatory and free radical scavenging activities of the isolated constituents from *M. zuihoensis*. These isolated constituents, quercetin (**1**) and the new compound, quercetin-3-*O*-β-D-glucopyranoside-(3′→*O*-3‴)-quercetin-3-*O*-β-D-galactopyranoside (**9**) exhibited markedly superoxide anion radical scavenging activities. Furthermore, quercetin (**1**), quercitrin (**3**), ethyl caffeate (**6**), ethyl 3-*O*-caffeoylquinate (**7**), and quercetin-3-*O*-β-D-gluco-pyranoside-(3′→*O*-3‴)-quercetin-3-*O*-β-D-galactopyranoside (**9**), showed various levels of inhibition of LPS-induced, early and late, inflammatory responses *in vitro*. Furthermore, this pharmacological property of the new compound **9** and other constituents isolated from *Machilus zuihoensis* may be advantageous in terms of its usage in suppression of oxidative stress and various related acute and chronic inflammatory disease, considering that a number of cellular inflammatory phenomena are orchestrated in onset of inflammatory diseases.
